# Intrinsically Negative Photosensitive Polyimides with Enhanced High-Temperature Dimensional Stability and Optical Transparency for Advanced Optical Applications via Simultaneous Incorporation of Trifluoromethyl and Benzanilide Units: Preparation and Properties

**DOI:** 10.3390/polym14183733

**Published:** 2022-09-07

**Authors:** Yanshuang Gao, Huasen Wang, Jie Jia, Zhen Pan, Xi Ren, Xinxin Zhi, Yan Zhang, Xuanzhe Du, Xiaolei Wang, Jingang Liu

**Affiliations:** 1 Beijing Key Laboratory of Materials Utilization of Nonmetallic Minerals and Solid Wastes, National Laboratory of Mineral Materials, School of Materials Science and Technology, China University of Geosciences, Beijing 100083, China; 2 POME Technology Co., Ltd., Liaocheng 252399, China

**Keywords:** photosensitive polyimide, photocrosslinking, optical transparency, coefficient of thermal expansion (CTE), benzanilide

## Abstract

Negative photosensitive polyimides (PSPIs) with the photo-patterned ability via the photocrosslinking reactions induced by the i-line (365 nm) and h-line (426 nm) emitting wavelengths in high-pressure mercury lamps have been paid increasing attention in semiconductor fabrication, optical fiber communications, and other advanced optoelectronic areas. In the current work, in view of the optical and thermo-mechanical disadvantages of the currently used negative PSPIs, such as the intrinsically photosensitive or auto-photosensitive systems derived from 3,3’,4,4’-benzophenonetetracarboxylic dianhydride (BTDA) and the ortho-alkyl- substituted aromatic diamines, a series of modified negative PSPIs with the enhanced optical transparency in the wavelength of 365~436 nm and apparently reduced coefficients of linear thermal expansion (CTE) were developed. For this purpose, a specific aromatic diamine with both of trifluoromethyl and benzanilide units in the molecular structures, 2,2’-bis(trifluoromethyl)-4,4’-bis[4-(4-amino-3-methyl)benzamide]biphenyl (MABTFMB) was copolymerized with BTDA and the standard 3,3’,5,5’-tetramethyl-4,4’-diaminodiphenylmethane (TMMDA) diamine via a two-step chemical imidization procedure. As compared with the pristine PI-1 (BTDA-TMMDA) system, the new-developed fluoro-containing PSPI systems (FPI-2~FPI-7) exhibited the same-level solubility in polar aprotic solvents, including N-methyl-2-pyrrolidone (NMP) and N,N- dimethylacetamide (DMAc). The FPI films cast from the corresponding FPI solutions in NMP showed the optical transmittances of 78.3–81.3% at the wavelength of 436 nm (*T*_436_, h-line), which were much higher than that of the PI-1 (*T*_436_ = 60.9%). The FPI films showed the CTE values in the range of 40.7 × 10^−6^/K to 54.0 × 10^−6^/K in the temperature range of 50 to 250 °C, which were obviously lower than that of PI-1 (CTE = 56.5 × 10^−6^/K). At last, the photosensitivity of the FPI systems was maintained and the micro-pattern with the line width of 10 μm could be clearly obtained via the standard photolithography process of FPI-7 with the molar ratio of 50% for MABTFMB in the diamine moiety.

## 1. Introduction

Photosensitive polyimides (PSPIs) represent a class of functional photoresists combining the functionalities of photosensitivity for common photoresists and the high-temperature and dielectric stability for polyimide dielectrics [1,2,3]. Thus, PSPIs have been widely used as passivation layers for semiconductor chips in microelectronic fabrication, interlayer dielectrics for flexible organic light-emitting diode (OLED) devices, or coatings for optical fibers [4,5,6]. It is different from the common photoresists which are usually used as the sacrifice medium for semiconductor chips that the PSPI layers are often contained in the final microelectronic or optoelectronic devices as permanent components. Thus, the PSPI components are often required to possess excellent combined properties, including high thermal stability, high mechanical properties, high dielectric properties, low dimensional shrinkage during thermal impact, low coefficients of thermal expansion, and sometimes high optical transmittance [7,8,9,10,11,12,13,14,15]. PSPIs could be roughly divided into two classes, including the positive PSPIs and negative ones depending on the different photochemistry mechanisms. For the former, the photo-decomposition reaction occurs in the exposure area, and so the same circuit pattern as the mask will be formed on the substrate after development. For the negative PSPIs, after exposure, the exposure parts are photo-crosslinked so that the opposite pattern of the mask is formed on the substrate after development. Both of the two kinds of PSPIs are usually consisted of photosensitive PI resins, photo active compounds (PACs), solvents, and other functional additives. Among the formulations of the PSPIs, the proportion of the PACs can usually reach 20 wt% or more so as to achieve enough photosensitivity for the PSPI systems in photolithography process [16]. After the pattern formation, the residual PACs have to be removed from the systems via post-baking process. Decomposition of the residual PACs might induce the undesirable shrinkage or thickness reduction of the pattern. Thus, the PSPIs with intrinsically photosensitive or auto-photosensitive features are highly desired in some specific applications.

Negative PSPI derived from 3,3’,4,4’-benzophenonetetracarboxylic dianhydride (BTDA) and ortho-alkyl-substituted aromatic diamines, such as 3,3’,5,5’-tetramethyl-4,4’-diaminodiphenyl- methane (TMMDA) was one of the most known auto-sensitive systems, and its photocrosslinking mechanism is based on the radical polymerization [17,18,19,20,21,22]. When the prebaked PSPI(BTDA-TMMDA) coating was exposed to high-pressure mercury lamp (365–436 nm), the benzophenone units will be excited to form the triplet, which will capture the hydrogen atom in the methyl substituents ortho to the amino groups in the diamine unit. Thus, the intra- and intermolecular photocrosslinking reactions occurred, making the exposed parts insoluble in the developer. This photoreaction efficiency was positively correlated with the numbers of alkyl substituents and benzophenone in the repeating units of the polymers. Additionally, the PSPI(BTDA-TMMDA) resin was soluble in polar aprotic solvents, such as N-methyl-2-pyrrolidone (NMP) and N,N-dimethylacetamide (DMAc), in the form of preimidized resin. Thus, the high-temperature imidization procedure with the processing temperature as high as 350 °C could be omitted for such a system, making it possible to form the passivation layer at relatively low temperature [23]. This is quite beneficial for specific temperature-sensitive applications, such as in flexible OLED fabrications [24]. The disadvantage of the negative auto-sensitive PSPI system is mainly reflected in two aspects. First, the optical transmittance of the PSPI(BTDA-TMMDA) to the emitting wavelength of high-pressure mercury lamp was relatively low due to the intrinsic molecular structure, so the photosensitivity of such system was a bit lower than that of the PAC-containing PSPI systems. Secondly, the CTE of the curing PSPI film is relatively high (56.5 × 10^−6^/K, 50–250 °C), which was not conducive to maintaining the dimensional stability of the polymer pattern at elevated temperature.

In our previous work [25], the rigid benzanilide unit was endeavored to incorporate into the molecular structure of the PSPI(BTDA-TMMDA) system via an aromatic diamine containing both of the benzanilide and ortho-substituted methyl groups in order to reduce the CTE value of the polymer. The CTE values as low as 42.0 × 10^−6^/K were achieved by such modification. However, incorporation of such benzanilide-containing diamine apparently sacrificed the optical transparency of the pristine PSPI system due to the UV and visible light absorption features of the rigid-rod benzanilide units. Deterioration of the optical transparency of the modified PSPI systems inevitably caused the reduction of the photosensitivity of the pristine polymer. 

Thus, in the current work, as one of continuous work on developing high-performance polyimides for high-tech applications, the intrinsically photosensitive PSPI systems with both high thermal-mechanical stability at elevated temperatures and good optical transparency or photosensitivity was developed. Effects of the trifluoromethyl and benzanilide units on the thermal, optical, and photosensitivity of the negative PSPI systems were investigated in detail.

## 2. Materials and Methods

### 2.1. Materials

UP grade of 3,3’,4,4’-benzophenonetetracarboxylic dianhydride (BTDA) was purchased from Evonik Degussa Corp. (Frankfurt, Germany) and dried in vacuum at 180 °C overnight prior to use. 3,3’,5,5’-Tetramethyl-4,4’-diaminodiphenylmethane (TMMDA) was purchased from Tokyo Chemical Industry (TCI) Co., Ltd. (Tokyo, Japan) and used as received. Furthermore, 2,2’-bis(trifluoromethyl)-4,4’-bis[4-(4-amino-3-methyl)benzamide]biphenyl (MABTFMB) was synthesized and characterized in our laboratory according to the procedures shown in the Supplementary files. It was purified by recrystallization from aqueous ethanol (InnoChem Sci. Technol. Co. Ltd. Beijing, China) before use. The diamine monomer was obtained as white powders with a purity of 99.6% according to the gas chromatography analysis. N-methyl-2-pyrrolidone (NMP), N,N-dimethylacetamide (DMAc), N,N-dimethyl-formamide (DMF), γ-butyrolactone (GBL), cyclopentanone (CPA), and other solvents were obtained from Tokyo Chemical Industry Co., Ltd. (Tokyo, Japan) and purified by distillation prior to use.

### 2.2. Characterization Methods

Inherent viscosities of the PI and FPI resins were measured using an Ubbelohde viscometer (Pingxuan Scientific Instrument Co., Ltd., Shanghai, China) with a 0.5 g dL^−1^ NMP solution at 25 °C. The number average molecular weight (M_n_) and weight average molecular weight (M_w_) of the PI and FPI resins were measured using a gel permeation chromatography (GPC) system (Shimadzu, Kyoto, Japan). HPLC-grade NMP was used as the mobile phase at a flow rate of 1.0 mL/min. Fourier-transform infrared (FTIR) spectra of the PI and FPI films were recorded on a Iraffinity-1S FT-IR spectrometer (Shimadzu, Kyoto, Japan). Ultraviolet-visible (UV-Vis) spectra of the PI and FPI films (thickness: ~20 μm) were recorded on a Hitachi U-3210 spectrophotometer (Tokyo, Japan) at room temperature. Wide-angle X-ray diffraction (XRD) was conducted on a Rigaku D/max-2500 X-ray diffractometer (Tokyo, Japan) with Cu-Kα1 radiation, operated at 40 kV and 200 mA. A JSM-6700F (JEOL, Tokyo, Japan) field emission scanning electron microscopy (FE-SEM) with an accelerating voltage of 15 kV was used to observe the micro-morphologies of the PI patterns. Yellow index (YI) values of the PI and FPI films were measured using an X-rite color i7 spectrophotometer (Grand Rapids, MI, USA) with PI samples at a thickness of 50 μm. The color parameters were recorded according to a CIE (International Commission on Illumination) Lab equation. L* is the lightness, where 100 means white and 0 implies black. A positive a* indicates a red color, and a negative one indicates a green color. A positive b* indicates a yellow color, and a negative one indicates a blue color. Thermogravimetric analysis (TGA) and the derivative TGA (DTG) of the PSPI films were performed on a Q50 thermal analysis system (New Castle, Delaware, USA) at a heating rate of 10 °C min^−1^ in nitrogen. Thermo-mechanical analysis (TMA) was recorded on a TMA402F3 thermal analysis system (NETZSCH, Selb, Germany). The thermal scanning mode ranges from 50 to 450 °C at a heating rate of 5 °C min^−1^ in nitrogen atmosphere. The coefficients of linear thermal expansion (CTE) values of composite films were recorded in the range of 50–250 °C.

Solubility was investigated by mixing 1.0 g of the PI and FPI resins and 9.0 g of the solvent tested (10 wt% solid content), and then stirred for 24 h at room temperature. The solubility was determined visually as three grades: completely soluble (++), partially soluble (+), and insoluble (−), wherein completely soluble indicates a homogenous and clean state without phase separation, precipitation or gel formation, and insoluble indicates no change of the resin in the appearance.

The photocrosslinking behaviors of the PSPIs were evaluated as follows. The PSPI varnish with a solid content of 15 wt% was spin-coated onto potassium bromide (KBr) sheets (1.0 cm × 1.0 cm × 2 mm) at 700 rpm for 30 s using a spin-coater (MS-B100, Mikasa Co. Ltd., Osaka, Japan). Then, KBr sheets were prebaked at 80 °C for 0.5 h on a digital hotplate (NA-1A, As-One Co., Ltd., Osaka, Japan). The thickness of the obtained PSPI coatings was 5 ± 0.5 μm. The KBr sheets were exposed to a UV light source (IMS-811A-0606, IUVOT Co., Ltd., Suzhou, China, power density: 400 mW/cm^2^). The distance between the UV source and the PSPI samples was 1.0 ± 0.1 cm. The UV exposure time was set to be 0.5 min, 1.0 min, 2.0 min, and 3.0 min. After exposure, the ATR-FTIR spectra of the PSPI samples were detected.

The patterning ability of the PSPIs was investigated by a photolithography procedure. Briefly, the PSPI varnish with a solid content of 15 wt% was spin-coated onto silicon wafer (diameter: 7.62 cm) at 700 rpm for 10 s and 3000 rpm for 30 s using a spin-coater (MS-B100, Mikasa Co., Ltd., Osaka, Japan). The wafer was prebaked at 120 °C for 100 s. Then, the PSPI was exposed for 60 s to a high-pressure mercury lamp equipped into a MA6 mask aligner (SUSS MicroTec, Garching, Germany) with the main ultraviolet wavelength of i-line (365 nm), g-line (405 nm), and h-line (436 nm). The total exposure dose was controlled to be 800 mJ cm^−2^. Then, the exposured PSPI was developed in DMF for 60 s, after which the wafer was immersed into butyl acetate for 60 s. At last, the PSPI was hard baked at 150 °C for 60 min and 250 °C for 60 min. The photolithography results were observed by SEM measurement. The thickness of the pattern was measured with Dektak XTL Stylus Profilometer (Tucson, AZ, USA).

### 2.3. PI synthesis and Film Preparation

The PI resins, including the standard PI-1 (BTDA-TMMDA) and the modified fluoro-containing FPI-2−FPI-7 were all synthesized via a two-stage chemical imidization procedure. The detailed synthesis procedure could be illustrated by the synthesis of FPI-7. At a yellow-light region in a clean room, the mixture diamine of TMMDA (3.8156 g, 0.015 mol), MABTFMB (8.7980 g, 0.015 mol) was added into the ultra-dry NMP (56.8 g) contained in a 250 mL three-necked flask equipped with a mechanical stirrer, a nitrogen inlet, and a cold water bath. In order to prevent the oxidation of the diamine monomer, dry nitrogen (purity ≥ 99.5%) was passed through the solution. Transparent diamine solution was then obtained after stirring at 10~15 °C for 30 min. Then, BTDA (9.6669 g, 0.03 mol) was added with an additional NMP (10.0 g) to give a reaction mixture with the solid content of 25 wt%. The cold water bath was removed and the temperature of the reaction mixture increased to room temperature (25 °C). The polymerization system was stirred at such temperature for 20 h to afford a highly viscous solution. Then, the dehydration system of acetic anhydride (Ac_2_O) (15.3 g, 0.15 mol) and pyridine (9.5 g, 0.12 mol) were added to the solution, and the reaction mixture was stirred at room temperature for another 24 h. The homogeneous PSPI solution was then successively poured into aqueous ethanol solution (500 mL, 75 vol%) to afford the filament resin. The precipitated FPI-7 resin was collected and dried at 100 °C in vacuum overnight. Yield of 20.6 g (97%) was achieved.

The FPI-7 resin was dissolved into DMAc with a solid content of 15 wt%. The homogeneous solution obtained was purified by filtration through a 0.50 μm Teflon syringe filter. Then, the FPI-7 varnish was spin-coated on a quartz substrate (diameter: 50 mm, TGK Co., Ltd., Tokyo, Japan). The thickness was controlled by adjusting the spinning rate. The coated substrate was thermally baked in an oven with the procedure of 80 °C/2 h, 120 °C/1 h, 180 °C/1 h, and 250 °C/1 h nitrogen. Then, the free-standing FPI-7 film was obtained after immersing the substrate into warm deionized water.

FPI-7. FTIR (cm^−1^): 1778, 1724, 1674, 1628, 1497, 1369, 1323, 1296, 1250, 1211, 1169, 1114, 860, and 725.

The other resins and films were prepared according to the similar procedure mentioned above with the formula shown in Table 1. It could be clearly seen that the molar ratio of the TMMDA/MABTFMB was set to be 100:0 for PI-1, 95:5 for FPI-2, 90:10 for FPI-3, 85:15 for FPI-4, 80:20 for FPI-5, 75:25 for FPI-6, and 50:50 for FPI-7, respectively.

PI-1. FTIR (cm^−1^): 1778, 1716, 1670, 1601, 1362, 1211, 1092, 849, and 721.

FPI-2. FTIR (cm^−1^): 1778, 1724, 1674, 1632, 1485, 1373, 1292, 1250, 1207, 1111, 833, and 729.

FPI-3. FTIR (cm^−1^): 1778, 1724, 1674, 1628, 1489, 1373, 1296, 1250, 1207, 1111, 833, and 729.

FPI-4. FTIR (cm^−1^): 1778, 1728, 1678, 1632, 1489, 1373, 1296, 1250, 1207, 1169, 1111, 833, and 725.

FPI-5. FTIR (cm^−1^): 1778, 1724, 1674, 1628, 1489, 1373, 1296, 1250, 1207, 1169, 1111, 833, and 725.

FPI-6. FTIR (cm^−1^): 1778, 1732, 1674, 1628, 1489, 1369, 1296, 1250, 1207, 1169, 1111, 860, and 725.

## 3. Results and Discussion

### 3.1. PI Synthesis and Film Preparation

**Figure 1:**
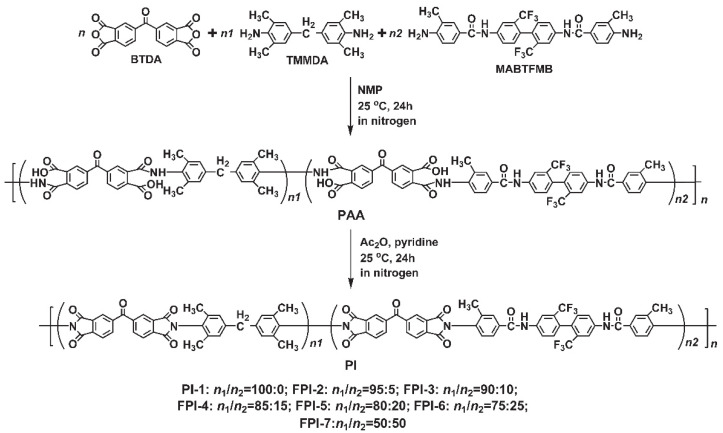
Preparation of standard photosensitive PI-1 (BTDA-TMMDA) and the fluoro- and benzamide-bridged FPI-2~FPI-7 resins.

The standard intrinsically PSPI, PI-1 (BTDA-TMMDA), together with several fluoro-modified FPI-2−FPI-7 with different contents of trifluoromethyl and benzamide units were designed and prepared via a two-step chemical imidization procedure, as shown in Figure 1. All the polymerization reactions were performed in the yellow-light region in order to avoid the undesirable photocrosslinking in the photosensitive varnishes. The entire polymerization system maintained homogeneous during the reaction, indicating the good solubility of the PI systems in the polymerization solvent. Even for FPI-7 with the molar ratio up to 50% for the rigid MABTFMB monomer in the total diamine systems, no gelling or precipitation were observed. The flexible and tough PI and FPI filaments were obtained after solution imidization. The inherent viscosities ([η]_inh_) and molecular weights of the obtained silky resins were measured and the results are listed in Table 2. It can be seen from the data that all the resins showed acceptable molecular weights for practical applications. For example, the resins showed [η]_inh_ values higher than 0.70 dL g^−1^, numerical molecular weights (M_n_) in the range of (3.85–5.06) × 10^4^ g mol^−1^ and polydispersity indices (PDI) around 2.0. This indicates that all the monomers showed good reactivity during the polymerization although the benzanilide-bridged diamine MABTFMB possessed lower reactivity than that of TMMDA, as could be deduced from the gradually decreased [η]_inh_ and M_n_ values of the polymers with the increasing contents of MABTFMB in the polymers. Nevertheless, the molecular weights of the current polymers could guarantee the following photolithography processing.

The MABTFMB diamine was designed with several structural features, including the ortho-substituted methyl groups in order to endow the derived PIs with photosensitivity and good solubility in organic solvents; rigid-rod benzanilide and biphenyl skeletons so as to reduce the CTE values of the derived PIs; and highly electronegative trifluoromethyl substituents in order to increase the optical transparency and solubility of the PIs. The solubility of the PI and FPI resins in common solvents was first detected and the results are shown in Table 2. As expected, all the PIs were soluble in polar aprotic solvents, such as NMP and DMAc. When the molar proportion of MABTFMB was lower than 10% (FPI-2 and FPI-3), the resins were soluble in cyclopentanone (CPA) at room temperature. FPI-2 was also soluble in THF. All the PIs were not soluble in γ-butyrolactone (GBL). Basically, with the increasing of the contents of MABTFMB in the polymers, the solubility gradually decreased, which is mainly due to the rigid benzanilide units in the MABTFMB moiety although the pendant methyl and trifluoromethyl substituents prohibited the compact packing of the molecular chains for the polymers. Nevertheless, the good solubility in NMP and DMAc makes it possible to fabricate the following film-forming and photolithography process with the preimidized forms of the current polymers. This is quite beneficial for decreasing the processing temperatures and reducing the thermal stress in the final coatings caused by the sharp thermal impact.

**Figure 2:**
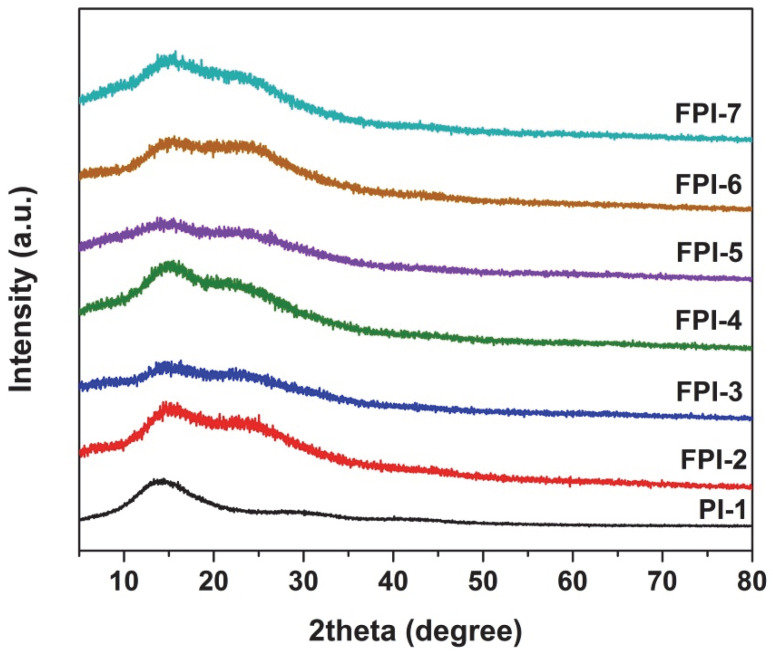
XRD spectra of PI and FPI polymers.

The good solubility of the current polymers could mainly be attributed to the synergic effects of the multi-substituted methyl and bulky trifluoromethyl groups in the backbones of the polymers. These substituents efficiently decreased the packing density of the molecular chains in the polymers although the rigid benzanilide units were prone to inducing the crystallinity in the polymer chains. This could be proven by the XRD measurements shown in Figure 2. All the PI films showed typical amorphous nature in the range of 5° to 80° for the scattering angles. Only broad peaks were found in the scattering angles range of 10–20°. Meanwhile, the PI-1 film showed the single absorption, while the FPI-2–FPI-7 exhibited the shoulder absorptions beside the main absorptions. This might be due to the co-polymeric nature of the FPI polymers. The repeating units of the BTDA-TMMDA and the BTDA-MABTFMB presented slightly different absorptions in the XRD spectra.

**Figure 3:**
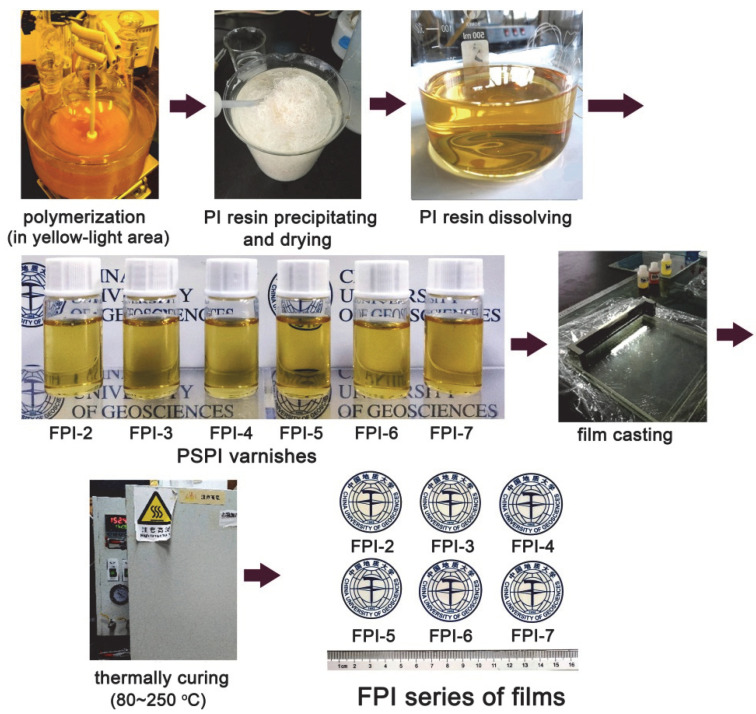
Preparation procedure for the photosensitive FPI series of films.

The good solution processability makes it possible to fabricate the PI films via a relatively low curing temperature process, as shown in Figure 3. First, the FPI solutions with the solid contents of 15 wt% were prepared by dissolving the corresponding resins in DMAc at room temperature. Then, the FPI solutions were cast onto clean glass substrates with a scraper. The thickness of the wet films was controlled by adjusting the gap between the scraper and the glass so as to afford the final cured PI films with the desired thickness. Then, the wet FPI films were cured in a nitrogen-purged oven with the curing temperature in the range of 80–250 °C. At last, the free-standing FPI films with the pale-yellow colors were obtained.

**Figure 4:**
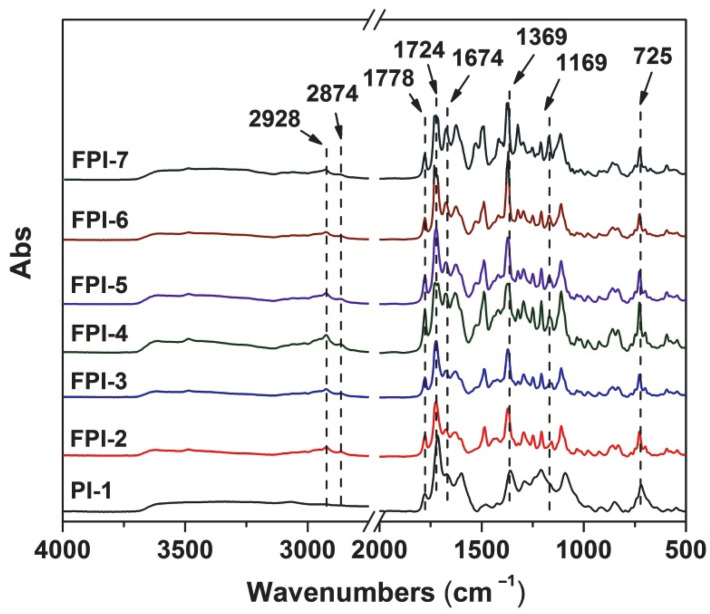
FTIR spectra of PI and FPI films.

The chemical structures of the PI and FPI film were detected by the FTIR measurement, which is shown in Figure 4. First, the characteristic absorptions of imide rings, including the peaks at 1778 cm^−1^ due to the asymmetrical carbonyl stretching vibrations, the peaks at 1724 cm^−1^ due to the symmetrical carbonyl stretching vibrations, the peaks at 1369 cm^−1^ assigned to the C–N stretching vibrations, and the peaks at 725 cm^−1^ due to the in-plane bending vibrations of carbonyl in imide rings were clearly observed. In addition, the characteristic absorptions at 1674 cm^−1^ due to the C=O in BTDA part were also detected. Meanwhile, the stretching vibrations at 2928 cm^−1^ and 2874 cm^−1^ assigned to the saturated C–H (–CH_2_– and –CH_3_) absorptions and the ones at 1489 cm^−1^ due to the stretching vibration of C=C in benzene ring were also detected. Finally, the characteristic absorptions at 1169 cm^−1^ due to the C–F bonds in MABTFMA units were only detected in FPI-2–FPI-7. The structural information is in good agreement with the anticipated structures for the polymers.

### 3.2. Thermal Properties

**Figure 5:**
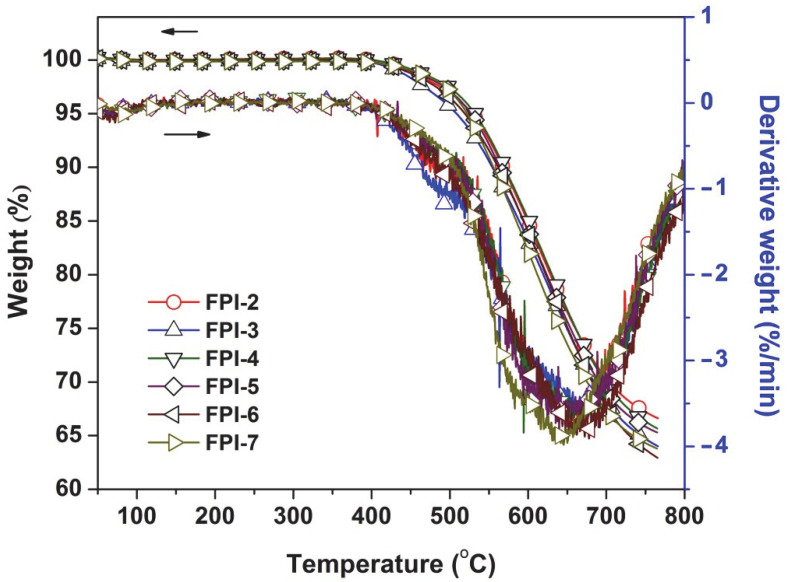
TGA and DTG curves of FPI films.

The standard negative auto-sensitive PSPI derived from BTDA and TMMDA (PI-1 in the current work) is known for the high thermal stability, including the high initial thermal decomposition temperatures (5% weight loss temperature, T_5%_ > 500 °C in nitrogen) and high glass transition temperatures (T_g_ > 340 °C) [17]. This is mainly attributed to the good thermal stability of benzophenone units in the dianhydride moiety and the sterically hindering effects of multi methyl groups in the diamine moiety. However, the PSPI (BTDA-TMMDA) presented a slightly higher CTE value (56.5 × 10^−6^/K, 50 × 250 °C) for practical applications. It can be anticipated that incorporation of MABTFMB components could maintain the intrinsic thermal stability of PSPI (BTDA-TMMDA) while efficiently decreased the CTE value of the polymer due to the existence of the rigid benzanilide and biphenyl units in the monomer. The thermal stabilities of the PI films were investigated by TGA and TMA measurements, respectively and the thermal data are summarized in Table 3. As could be seen from the TGA plots of the polymers shown in Figure 5 that the FPI films showed T_5%_ and R_w750_ values in the range of 509–532 °C and 64–67wt%, respectively, which were close to those of PI-1 (T_5%_ = 532 °C; R_w750_ = 72%). For the FPI copolymers, the maximum thermal decomposition occurred in the temperature range of 600~700 °C, as could be observed from the derivative TGA (DTG) plots in Figure 5. Thus, the current modified polymers showed excellent thermal stability.

**Figure 6:**
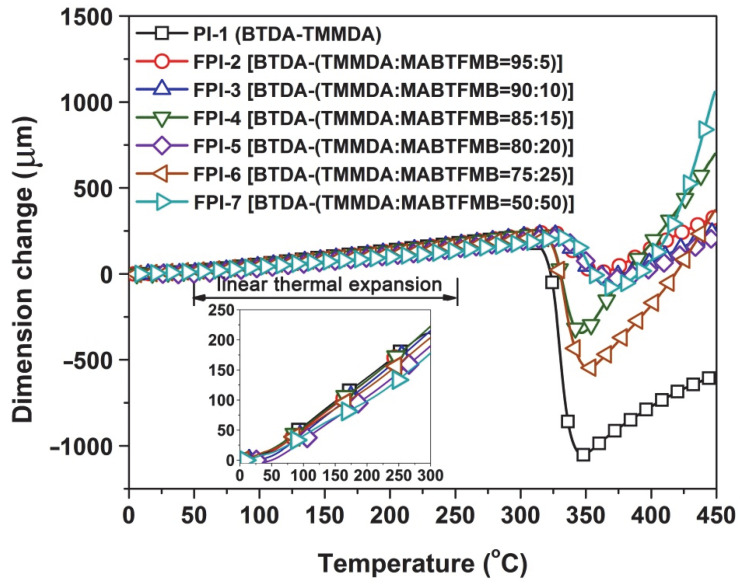
TMA curves of PI and FPI films.

Although the currently developed FPI copolymers showed similar thermal stability with the pristine PI-1 (BTDA-TMMDA), the high-temperature dimensional stability was obviously increased. This could be deduced from the TMA plots of the polymers shown in Figure 6. First, it could be clearly observed that all the polymers exhibited thermally shrinking behaviors when the test temperatures were higher than T_g_ values of the polymers. For the high-temperature-resistant polymers, this high-temperature shrinkage beyond the T_g_ is usually considered to be a manifestation of the orderly arrangement of the molecular chain segments in the polymers. When the ordered arrangements finish, the dimension of the polymers will continuously expand with the increasing test temperature. The turning points of this dimensional change are usually recorded as the T_g_ values of the polymers. According to the records, the FPI copolymers showed the T_g_ values in the range of 348.0–370.6 °C, which were all higher than that of PI-1(BTDA-TMMDA) (T_g_ = 347.6 °C). It meant that the copolymers showed enhanced high-temperature dimensional stability after incorporation of MABTFMA components. This enhancement could be further quantitatively reflected by the CTE values of the polymers, as shown in Table 3. As expected, the CTE values of the polymers gradually decreased with the increasing contents of MABTFMB in the polymers. The FPI films showed reduced CTE values in the range of 40.7 × 10^−6^/K to 54.0 × 10^−6^/K in the temperature range of 50–250 °C. Undoubtedly, the improvement of the thermally dimensional stability of the copolymers could mainly be attributed to the rigid benzanilide and biphenyl units in the polymers. Considering the solution processability of the current copolymers, the CTE values as low as 40.7 × 10^−6^/K could efficiently expand their applications in advanced optoelectronic fabrications.

### 3.3. Optical Properties

As mentioned before, there are two main targets for the current work. The first is to improve the high-temperature dimensional stability (decreasing CTE value) of standard PSPI(BTDA-TMMDA) system, and the second one is to increase the optical transparency of the system to i-line (365 nm), g-line (405 nm), and h-line (436 nm) of high-pressure mercury lamp sources. Both targets are based on the prerequisite that the photosensitivity of the pristine PSPI (BTDA-TMMDA) system should be maintained or increased for better. According to the thermal property evaluation results, the CTE value of the system could be obviously decreased via incorporation of MABTFMB components. Thus, the effects of introduction of MABTFMB on the optical properties, including optical transparency, photocrosslinking, and photolithographic features, were investigated.

**Figure 7:**
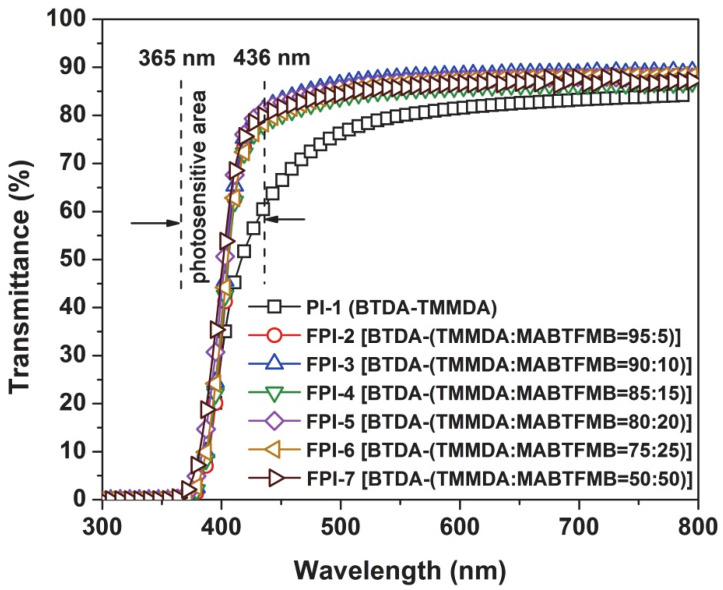
UV-Vis spectra of PI and FPI films.

First, the ultraviolet-visible (UV-Vis) spectra of the FPI films with a thickness of 20 μm were tested and the results are shown in Figure 7 and Table 3. It can be clearly observed that in the wavelength range of 365 to 436 nm, all the FPI films exhibited much better optical transmittances than that of the pristine PI-1 although they showed the similar cutoff wavelength (λ_cut_). For example, the FPI-7 film should the optical transmittance value of 58.2% at 405 nm (T_405_) and 81.3% at 436 nm (T_436_), which were obviously higher than those of PI-1 (T_405_ = 38.1%; T_436_ = 60.9 %). It is well established that the optical transparency of PI films is highly related to the charge transfer (CT) interactions in the molecular structures, in which the diamine units act as the electron donors and the dianhydride units as the electron acceptors [26,27,28]. The stronger the intra- and intermolecular CT interactions in the PIs, the stronger the absorption of the visible light by the PI films, and the deeper the color of the PI films. Thus, it is essential to eliminating or prohibiting the CT interactions in order to increase the optical transmittances of the PI films. In the current work, the incorporation of the highly electronegative trifluoromethyl (–CF_3_) undoubtedly prohibited the CT interactions in the copolymers, thus endowing the FPI films superior optical transparency. The superior optical properties could further be reflected by the CIE Lab optical parameters, which are shown in Table 3. Basically, the lightness (L*) of the PI films gradually increased and the yellowness (b*) decreased with the increasing of the contents of MABTFMB components in the polymers. For instance, FPI-7 film showed L* and b* values of 97.92 and 1.27, respectively, which are obviously better than those of the pristine PI-1 (L* = 93.68; b* = 16.92). Furthermore, incorporation of the MABTFMB components decreased the haze values of the copolymer films. The improvement of optical parameters of the PI films is quite beneficial for the following photocrosslinking and photolithographic processing.

**Figure 8:**
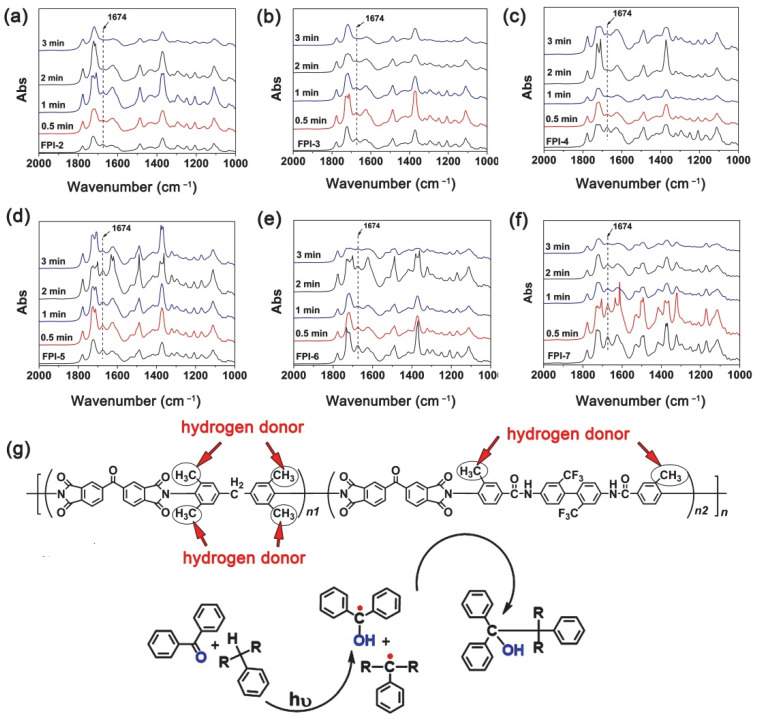
Changes of the absorption strength of benzophenone carbonyl at 1674 cm^−1^ with the exposure time (high-pressure mercury lamp, power: 400 mW/cm^2^). (**a**) FPI-2; (**b**) FPI-3; (**c**) FPI-4; (**d**) FPI-5; (**e**) FPI-6; (**f**) FPI-7; (**g**) Photocrosslinking mechanisms.

Then, effects of the incorporation of MABTFMB components on the photocrosslinking reactivity of the PSPI films were detected, and the results are shown in Figure 8a–f. The characteristic absorption strength of benzophenone carbonyl groups at the wavenumber of 1674 cm^−1^ in FTIR spectra were used as the indicator for the UV-induced photoreactions. If the carbonyl groups were excited to be triplets, the absorption strength at 1674 cm^−1^ will decrease. The UV exposure times were set to be 0.5 min, 1 min, 2 min, and 3 min, respectively. It can be clearly observed that all the copolymers could undergo the UV excitation reaction upon exposure and most of the photoreactions finished within 3 min. In our previous study, the PI-1 (BTDA-TMMDA) system has been proven to be able to finish the reaction within 2 min under the same conditions according to the photocrosslinking mechanism shown in Figure 8g [25]. Thus, incorporation of MABTFMB slightly deteriorated the photosensitivity of the pristine PI-1. It has been proven that the efficiency of photocrosslinking in such PSPI systems was proportional to the square of alkyl substituents [17]. Thus, the deterioration of the photosensitivity in the copolymers might be due to the lower mass fractions of methyl groups in MABTFMB compared with those in TMMDA. Nevertheless, the two methyl groups in the repeating units of the BTDA-MABTFMB moiety guaranteed the acceptable photosensitivity for many practical applications. The future work might be focused on the interfaces between the current polymers and the substrates [29].

**Figure 9:**
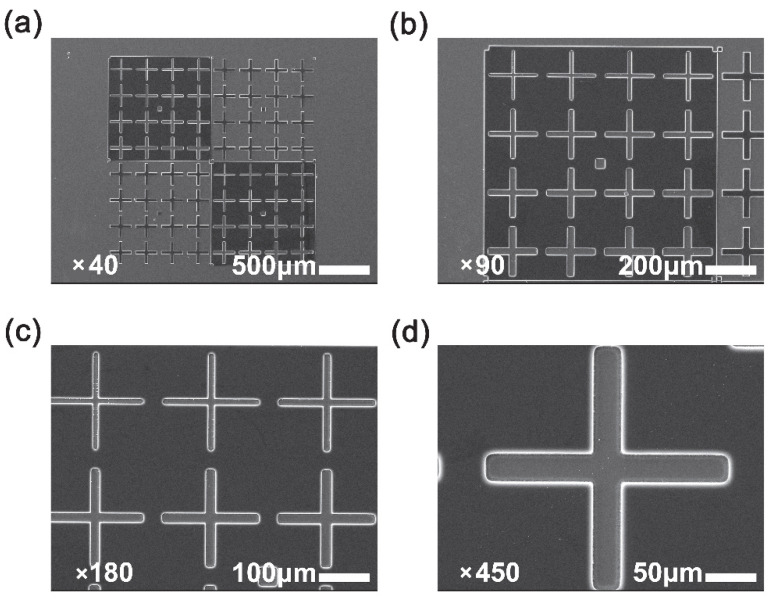
SEM photos of micro-patterns with different magnification formed by the photolithography process of FPI-7. (**a**) magnification times: 40 (×40); (**b**) magnification times: 90 (×90); (**c**) magnification times: 180 (×180); (**d**) magnification times: 450 (×450).

Finally, the photolithographic behaviors of the modified PSPI copolymers were investigated by representative FPI-7. According to the photolithographic procedure shown in Section 2.2, the pattern was fabricated and the results are shown in Figure 9. It can be observed that clear cross-shaped pattern with the line width around 20–30 μm and thickness around 6–8 μm (measured by stylus Profilometer) could be formed by the photolithography procedure. Meanwhile, the pattern showed good adhesion to the silicon wafer and no peeling or warpage occurred during the developing and rinsing process. This might be due to the reduced CTE of FPI-7 (40.7 × 10^−6^/K) film. The mismatch of CTE values between the polymer coatings and silicon wafer (3–4 × 10^−6^/K) was thought to be one of the most important factors causing the fractures or cracks in the semiconductor structures [30]. Thus, the reduction of the CTE value of the current FPIs is helpful for reducing the buildup of the mechanical stress and thus improving the reliability of the optoelectronic devices. 

## 4. Conclusions

Rigid benzanilide and biphenyl units and highly electronegative trifluoromethyl groups were incorporated into the molecular structure of auto-sensitive PI-1 (BTDA-TMMDA) via the copolymerization with MABTFMB diamine in order to simultaneously improve the optical transparency and high-temperature dimensional stability of the pristine polymer while maintain the intrinsic photosensitivity. Various characterizations proved the successful achievement of the molecular design. The FPI copolymers exhibited good combined thermal and optical properties. The FPI-7 film containing 50 mol% MABTFMB component in the diamine units exhibited the best comprehensive properties, including good solution processability, a CTE value of 40.7 × 10^−6^/K in the temperature range of 50–250 °C, a T_5%_ value of 522 °C, a T_g_ value of 370.6 °C, a T_405_ value of 58.2%, a T_436_ value of 81.3%, a yellow index (b*) of 1.27, and good photoimageable properties. The optical transparency and CTE values of the FPI-7 were all better than those of the PSPI-4 polymer without fluoro-containing substituents reported in our previous work (T_405_ = 15.2%, CTE = 43.0 × 10^−6^/K) [25]. Good properties make the FPI films good candidates for advanced optoelectronic applications.

## Figures and Tables

**Table 1:** Formula for the PI and FPI synthesis.

PI	BTDA (g, mol)	TMMDA (g, mol)	MABTFMB (g, mol)	NMP (g)	Ac_2_O (g, mol)	Pyridine (g, mol)
PI-1	9.6669, 0.03	7.6311, 0.03	0	69.2	15.3, 0.15	9.5, 0.12
FPI-2	9.6669, 0.03	7.2495, 0.0285	0.8798, 0.0015	53.4	15.3, 0.15	9.5, 0.12
FPI-3	9.6669, 0.03	6.8680, 0.0270	1.7596, 0.0030	54.9	15.3, 0.15	9.5, 0.12
FPI-4	9.6669, 0.03	6.4864, 0.0255	2.6394, 0.0045	56.4	15.3, 0.15	9.5, 0.12
FPI-5	9.6669, 0.03	6.1049, 0.0240	3.5192, 0.0060	57.9	15.3, 0.15	9.5, 0.12
FPI-6	9.6669, 0.03	5.7233, 0.0225	4.3990, 0.0075	59.4	15.3, 0.15	9.5, 0.12
FPI-7	9.6669, 0.03	3.8156, 0.0150	8.7980, 0.0150	66.8	15.3, 0.15	9.5, 0.12

**Table 2:** Inherent viscosities, molecular weights, and solubility of SPI resins.

PI	[η]_inh_ ^a^ (dL g^−1^)	Molecular Weight ^b^			Solubility ^c^				
		M_n_ (×10^4^ g mol^−1^)	M_w_ (×10^4^ g mol^−1^)	PDI	NMP	DMAc	GBL	CPA	THF
PI-1	1.00	5.06	8.98	1.77	++	++	−	++	++
FPI-2	0.96	4.89	9.19	1.88	++	++	−	++	++
FPI-3	0.92	4.80	9.16	1.91	++	++	−	++	+
FPI-4	0.91	4.57	8.83	1.93	++	++	−	+	−
FPI-5	0.88	4.29	8.06	1.88	++	++	−	−	−
FPI-6	0.86	4.26	7.95	1.87	++	++	−	−	−
FPI-7	0.77	3.85	7.55	1.96	++	++	−	−	−

^a^ Inherent viscosities measured with a 0.5 g dL^−1^ PI solution in NMP at 25 °C; ^b^ M_n_: number average molecular weight; M_w_: weight average molecular weight; PDI: polydispersity index, PDI = M_w_/M_n_; ^c^ ++: Soluble; +: partially soluble; −: insoluble. NMP: N-methyl-2-pyrrolidone; DMAc: N,N-dimethylacetamide; GBL: γ-butyrolactone; CPA: cyclopentanone; THF: tetrahydrofuran.

**Table 3:** Thermal and optical properties of PI and FPI films.

PI	Thermal Properties ^a^				Optical Properties ^b^						
	T_g_ (°C)	T_5%_ (°C)	R_w750_ (%)	CTE (×10^−6^/K)	λ_cut_ (nm)	T_405_ (%)	T_436_ (%)	L*	a*	b*	Haze (%)
PI-1	347.6	530	72	56.5	359	38.1	60.9	93.68	−2.21	16.92	4.31
FPI-2	360.7	528	67	54.0	357	47.3	78.3	94.99	−1.18	4.52	1.51
FPI-3	367.6	509	65	53.9	359	48.1	78.6	94.97	−1.12	4.61	1.38
FPI-4	348.0	532	66	52.5	364	49.5	79.9	94.97	−0.81	3.59	1.43
FPI-5	348.7	527	66	48.5	364	51.3	80.1	95.07	−1.04	4.03	0.45
FPI-6	351.7	519	64	47.5	366	55.6	81.1	95.20	−1.13	4.24	0.72
FPI-7	370.6	522	64	40.7	367	58.2	81.3	97.92	−0.29	1.27	1.33

^a^ T_g_: Glass transition temperature; T_5%_: Temperatures at 5% weight loss; R_w750_: Residual weight ratio at 750 °C in nitrogen; CTE: linear coefficient of thermal expansion in the range of 50–250 °C. ^b^ λ_cut_: Cutoff wavelength; T_405_, T_436_: Transmittance at the wavelength of 405 nm and 436 nm with a thickness of 20 μm, respectively; L*, a*, b*, see Measurements part.

## Data Availability

Data is contained within the article.

## Supplementary Materials

The following supporting information can be downloaded at: https://www.mdpi.com/article/10.3390/polym14183733/s1, Supplementary File: the Synthesis and Characterization of 2,2’-bis(trifluoromethyl)-4,4’-bis[4-(4-nitro-3-methyl)benzamide] biphenyl(MNBTFMB) and 2,2’-bis(trifluoromethyl)-4,4’-bis[4-(4-amino-3-methyl)benzamide] biphenyl(MABTFMB).
