# Supplementary material for: Intrinsically Negative Photosensitive Polyimides with Enhanced High-Temperature Dimensional Stability and Optical Transparency for Advanced Optical Applications via Simultaneous Incorporation of Trifluoromethyl and Benzanilide Units: Preparation and Properties
Source: Polymers (Basel). 2022 Sep 7;14(18):3733. doi: 10.3390/polym14183733 (PMC9503198; doi:10.3390/polym14183733)
Supplement: Supplementary file 1 [file polymers-14-03733-s001.zip › polymers-1872360-supplementary.pdf]

polymers

MDPI

Article

Intrinsically negative photosensitive polyimides with enhanced high-temperature dimensional stability and optical transparency for advanced optical applications via simultaneous incorporation of trifluoromethyl and benzanilide units: Preparation and properties

Yanshuang Gao [1†], Huasen Wang [2†], Jie Jia [2†], Zhen Pan [1], Xi Ren [1], Xinxin Zhi [1], Yan Zhang [1], Xuanzhe Du [1], Xiaolei Wang [1] and Jingang Liu [1*]

[1] Beijing Key Laboratory of Materials Utilization of Nonmetallic Minerals and Solid Wastes, National Laboratory of Mineral Materials, School of Materials Science and Technology, China University of Geosciences, Beijing 100083, China; 2103210035@cugb.edu.cn (Y.G.); 2103210036@cugb.edu.cn (Z.P.); renxi415@hotmail.com (X.R.); 3003200015@cugb.edu.cn (X.Z.); 3003200016@cugb.edu.cn (Y.Z.); 1003200609@cugb.edu.cn (X.D.); xiaoleiwang2022@163.com (X.W.)

[2] POME Technology Co., Ltd., Liaocheng, 252399, China; whs@pome.com.cn (H.W.); jiajie@pome.com.cn (J.J.)

* Correspondence: liujg@cugb.edu.cn; Tel.: +86-10-82322972

† These authors contributed equally to this work.

Citation: Gao, Y.; Wang, H.; Jia, J.; Pan, Z.; Ren, X.; Zhi, X.; Zhang, Y.; Du, X.; Wang, X.; Liu, J. Intrinsically negative photosensitive polyimides with enhanced high-temperature dimensional stability and optical transparency for advanced optical applications via simultaneous incorporation of trifluoromethyl and benzanilide units: Preparation and properties. *Polymers* **2022**, *14*, 3733. https://doi.org/10.3390/polym14183733

Academic Editor: Hao-Wen Cheng

Received: 2 August 2022
Accepted: 4 September 2022
Published: 7 September 2022

Publisher's Note: MDPI stays neutral with regard to jurisdictional claims in published maps and institutional affiliations.

Copyright: © 2022 by the authors. Licensee MDPI, Basel, Switzerland. This article is an open access article distributed under the terms and conditions of the Creative Commons Attribution (CC BY) license (https://creativecommons.org/licenses/by/4.0/).

Supplementary Files:

Materials

3-Methyl-4-nitrobenzoyl chloride (MNBC), anhydrous tetrahydrofuran (THF), 5% palladium on active carbon (Pd/C), hydrazine monohydrate ($NH_2NH_2{\cdot}H_2O$), anhydrous ethanol were purchased from Beijing InnoChem Science & Technology Co., Ltd. (Beijing, China) and used as received. 2,2'-Bis(trifluoromethyl)-4,4'-biphenyl (TFMB) was purchased from Tokyo Chemical Industry (TCI) Co., Ltd. (Tokyo, Japan) and used as received.

Characterization methods

Melting points of the chemical compounds were measured by differential scanning calorimetry (DSC) on a TA-Q 100 thermal analysis system (New Castle, Delaware, USA) at a heating rate of 10 °C min^{-1} in nitrogen. Fourier transform infrared (FTIR) spectra of the monomers were recorded on an Iraffinity-1S FT-IR spectrometer (Shimadzu, Kyoto, Japan). Nuclear magnetic resonances (^{1}H-NMR and ^{13}C-NMR) were performed on an AV 400 spectrometer (Ettlingen, Germany) operating at 400 MHz in deuterated dimethyl sulfoxide (DMSO-d_6). Electrospray ionization mass spectrometry (ESI-MS) was performed on an Esquire 3000 mass spectrometer (Bruker Daltonik, Bremen, Germany). Elemental analysis was performed on a Thermo Eager 300 Flash EA1112 elemental analyzer (Waltham, MA, USA).

Synthesis of 2,2'-bis(trifluoromethyl)-4,4'-bis[4-(4-nitro-3-methyl)benzamide] biphenyl (MNBTFMB)

In a representative synthesis procedure, a 1000-mL three-necked flask fitted with a magnetic stirrer, a clod bath, a thermometer and a dropping funnel was added TFMB (128.1 g, 0.40 mol), anhydrous THF (300 mL), and anhydrous pyridine (123.5 g, 1.56 mol). The mixture was cooled to −5 °C and the solution of 3-methyl-4-nitrobenzoyl chloride (MNBC, 199.6 g, 0.10 mol) dissolved in THF (500 mL) was added dropwisely over a period

of 1.0 h. Then, the cold bath was removed and the reaction mixture was stirred at room temperature for another 24 h. Then, the mixture was poured into an excess amount of aqueous potassium carbonate solution with a concentration of 10 wt%. The precipitate was collected by filtration, washed with water, and dried in vacuo at 100 °C for 24 h. The obtained white powder was recrystallized from 2-methoxyethanol to afford pale-yellow needle crystals (MNBTFMB). Yield: 181.0 g (70.0%). FTIR (KBr, cm^{-1}): 2936, 1659, 1593, 1524, 1489, 1416, 1327, 1169, and 1119. Nuclear magnetic resonance (^{1}H-NMR) (400 MHz, DMSO-d_6, ppm): 10.85 (s, 2H), 8.33 (s, 2H), 8.16–8.09 (m, 4H), 7.96 (d, 2H), 7.43–7.41 (d, 2H), and 2.61 (s, 6H). ^{13}C-NMR (100 MHz, DMSO-d_6, ppm): 164.9, 162.8, 151.2, 139.6, 138.8, 133.3, 133.1, 132.6, 132.2, 127.2, 125.0, 123.1, 117.7, and 19.8. ESI-MS: m/z=645.30 (M^+). Elemental analysis: calculated for $C_{30}H_{20}F_6N_4O_6$ (M_w: 646.49 g/mol): C, 55.73 %, H, 3.12 %, N, 8.67 %; found: C, 55.46 %, H, 3.38%, N, 8.59%.

Synthesis of 2,2'-bis(trifluoromethyl)-4,4'-bis[4-(4-amino-3-methyl)benzamide] biphenyl (MABTFMB)

A 1000-mL three-necked flask fitted with a magnetic stirrer, a thermometer and a dropping funnel was charged with a mixture of MNBTFMB (30.0 g, 46.4 mmol), absolute ethanol (250 mL) and a catalytic amount of 5% palladium on activated carbon (3.0 g). The reaction mixture was heated to reflux and then hydrazine monohydrate (80 mL, 1.648 mol) diluted with ethanol (50 mL) was added dropwisely over a period of 2.0 h. After the addition was completed, the reaction system was refluxed for 6 h. Then the hot mixture was filtered to remove the catalyst and the filtrate was cooled to room temperature. The precipitated white powders were filtered out, washed with cold ethanol and dried under vacuo at 80 °C overnight to afford BAPPT. Yield: 14.2 g (52.2%). Purity (high-performance liquid chromatography, HPLC): 99.5%. Melting point: 275.9 °C (DSC peak temperature). FTIR (KBr, cm^{-1}): 3499, 3402, 2928, 1647, 1624, 1582, 1528, 1504, 1412, 1315, 1254, and 1173. Nuclear magnetic resonance (^{1}H-NMR) (400 MHz, DMSO-d_6, ppm): 10.13 (s, 2H), 8.32 (s, 2H), 8.09–8.07 (d, 2H), 7.68–7.64 (m, 4H), 7.32–7.30 (d, 2H), 6.69–6.67 (d, 2H), 5.59 (s, 4H), and 2.14 (s, 6H). ^{13}C-NMR (100 MHz, DMSO-d_6, ppm): 166.3, 151.1, 140.5, 132.8, 131.1, 130.6, 128.2, 128.0, 127.6, 125.8, 123.1, 122.5, 121.1, 120.3, 117.2, 113.1, and 18.0. ESI-MS: m/z=587.4 (M^+), 609.4 $(M+23)^+$, and 625.3 $(M+39)^+$. Elemental analysis: calculated for $C_{30}H_{24}F_6N_4O_2$ (M_w: 586.53 g/mol): C, 61.43%, H, 4.12%, N, 9.55%; found: C, 61.27%, H, 4.12%, N, 9.51%.

Results and Discussion

The fluoro-containing aromatic diamine MABTFMB was synthesized by a two-step procedure shown in **Figure S1**. 3-Methyl-4-nitrobenzoyl chloride (MNBC) was first reacted with TFMB at low temperature in the solvent to afford the dinitro compounds MNBTFMB. Pyridine was used as the absorbent for the hydrogen chloride (HCl) by-products. Then, MNBTFMB was reduced by hydrazine under the catalysis of Pd/C to afford the target diamine monomer with a moderate yield.

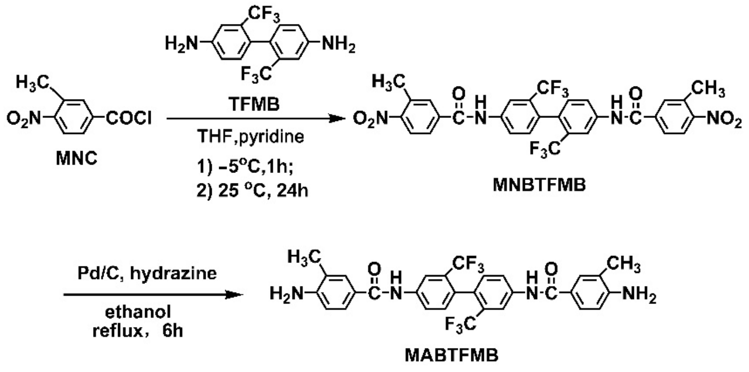

Figure S1. Synthesis procedure for MABTFMB.

The chemical structures of MABTFMB and the MNBTFMB precursor were detected. **Figure S2** shows the FTIR spectra of the compounds with the assigned characteristic absorption peaks. It can be clearly observed that the characteristic absorptions of $–NO_2$ in MNBTFMB at 1593 cm^{-1} and 1327 cm^{-1} ascribed to the asymmetrical and symmetrical stretching vibrations, respectively, totally disappeared in the spectrum of MABTFMB. Instead, the new absorptions at 3499 cm^{-1} and 3402 cm^{-1} due to the N–H stretching of primary amino groups appeared in the spectrum of MABTFMB. Meanwhile, the characteristic absorptions at 1647 cm^{-1} due to the stretching vibration of C=O in the amide (–CONH–) units, the one at 1504 cm^{-1} due to the stretching vibration of C=C in benzene ring, the one at 1254 cm^{-1} due to the stretching vibration of C–O–C, and the one at 1173 cm^{-1} due to the stretching vibration of C–F were all clearly observed.

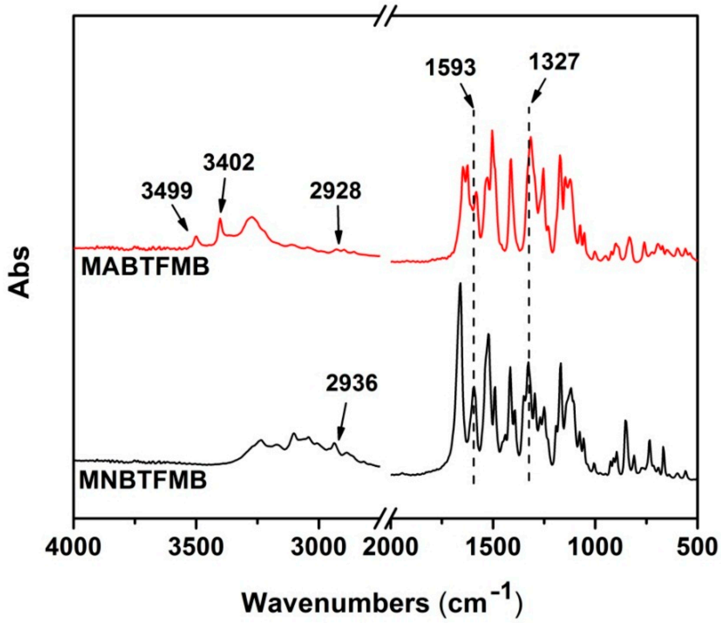

Figure S2. FTIR spectra of MNBTFMB and MABTFMB.

Figure S3 presents the ^1H-NMR spectra of MABTFMB and MNBTFMB, respectively. In the ^1H-NMR spectra, for both of the compounds, the H protons in the amide (–CONH–) units showed the absorption at the farthest downfield in the spectrum, while those in the methyl ($–CH_3$) groups showed the absorptions at the farthest upfield in the spectrum except the solvent peaks. For the diamine of MABTFMB (Figure S3a), the amino groups showed clear absorption at the chemical shift of 5.59 ppm. The proton of H_b exhibited the absorption at the second farthest downfield in the spectrum of MNBTFMB due to the electron-withdrawing nature of the ortho-substituted nitro groups. However, the absorption of H_b in MABTFMB moved to the upfield area in the spectrum of MABTFMB due to the electron-donating nature of the ortho-substituted amino groups. The phenomenon is in good agreement with the expected structural features of the MABTFMB and the dinitro intermediate.

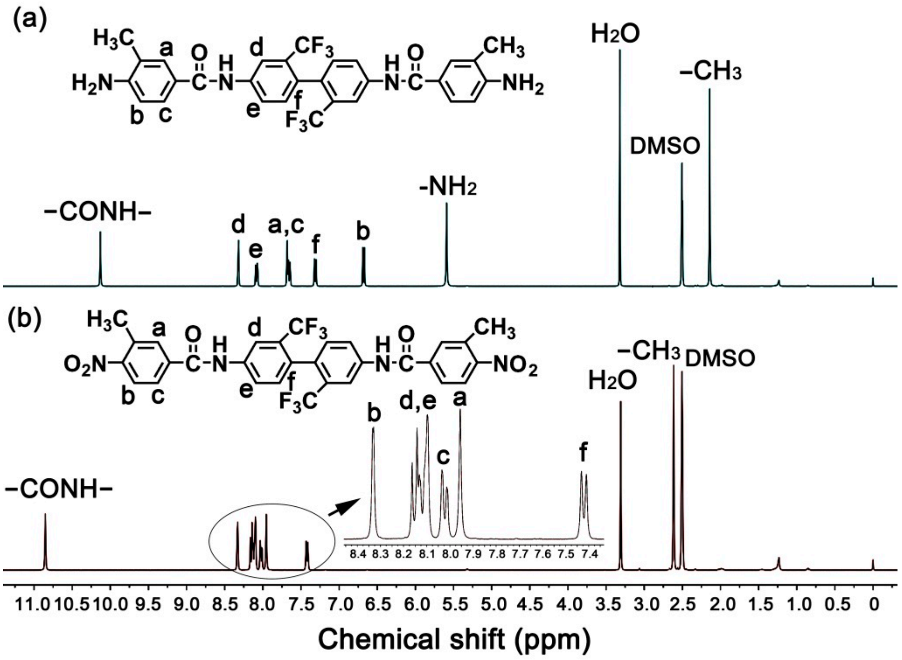

Figure S3. ^{1}H-NMR spectra of MABTFMB (**a**) and MNBTFMB (**b**).

As for the ^{13}C-NMR measurements of MABTFMB, it could be clearly seen form the spectra shown in Figure S4 that 15 signals were recorded in the ^{13}C-NMR spectrum (Figure S4b), in which, only 7 carbon signals with protons attached were detected in the distortionless enhancement by polarization transfer (DEPT-135) spectrum (Figure S4a).. The methyl carbon (–CH_3) showed the absorptions at the farthest upfield in the spectra of MABTFMB, while the C_7 in the amide (–CONH–) exhibited the absorption at the farthest downfield in the spectra due to the electron-withdrawing nature of the carbonyl groups. In addition, C_{10} and C_{11} showed clear quartet absorptions in the chemical shift range of 110–135 ppm, respectively, which was due to the $^{2}J_{C\text{-}F}$ and $^{3}J_{C\text{-}F}$ coupling of the carbons with the fluorine atoms in the diamine. All the information was consistent with the expected structure of the target MABTFMB diamine.

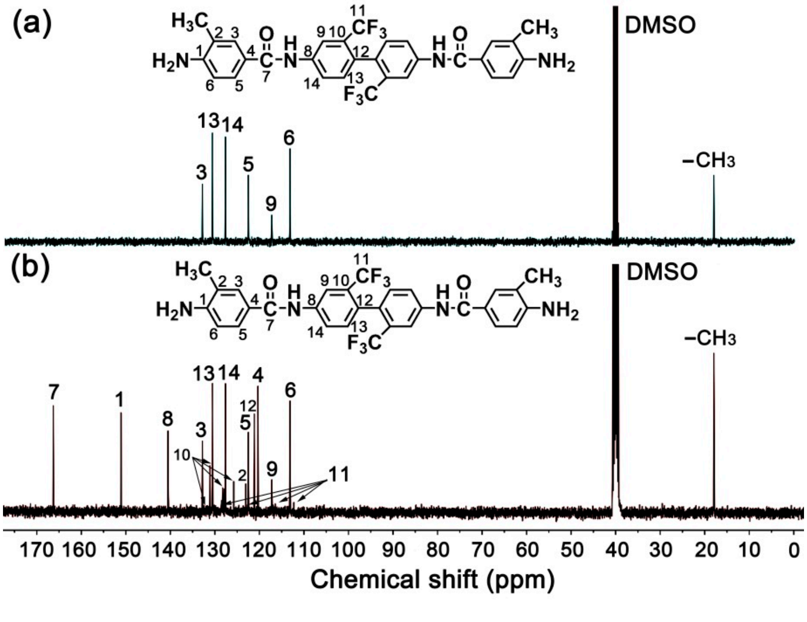

Figure S4. ^{13}C-NMR spectra of MABTFMB. (a) DEPT-135; (b) ^{13}C-NMR.

At last, the elemental analysis results also revealed the successful preparation of the target diamine. The characterization results demonstrated that the MABTFMB diamine was successfully synthesized and could be used for the following polymerization.
